# Analyzing Insights of Super-Response in Cardiac Resynchronization Therapy with Fusion Pacing

**DOI:** 10.3390/diagnostics15091118

**Published:** 2025-04-28

**Authors:** Alexandra-Iulia Lazăr-Höcher, Simina Crișan, Cristina Văcărescu, Samuel Nistor, Adelina Andreea Faur-Grigori, Andreea Cozgarea, Petru Baneu, Liviu Cirin, Laurențiu Brăescu, Larissa Dăniluc, Dan Gaiță, Constantin-Tudor Luca, Dragoș Constantin Cozma

**Affiliations:** 1 Doctoral School, “Victor Babeș” University of Medicine and Pharmacy, 300041 Timișoara, Romania; alexandra.hocher@umft.ro (A.-I.L.-H.); andreea.faur@umft.ro (A.A.F.-G.); andreea.cozgarea@umft.ro (A.C.); petru.baneu@umft.ro (P.B.); liviu.cirin@umft.ro (L.C.); larissa.daniluc@umft.ro (L.D.); 2 Institute of Cardiovascular Diseases Timisoara, 300310 Timisoara, Romania; cristina.vacarescu@umft.ro (C.V.); samuel.nistor@umft.ro (S.N.); laurentiu.braescu@umft.ro (L.B.); dan.gaita@umft.ro (D.G.); constantin.luca@umft.ro (C.-T.L.); dragos.cozma@umft.ro (D.C.C.); 3 Research Center of the Institute of Cardiovascular Diseases Timisoara, 300310 Timisoara, Romania; 4 Department of Cardiology, “Victor Babeș” University of Medicine and Pharmacy, 300041 Timisoara, Romania; 5 Center for Modeling Biological Systems and Data Analysis, “Victor Babeș” University of Medicine and Pharmacy, 300041 Timișoara, Romania; 6 Emergency County Clinical Hospital Sibiu, 550245 Sibiu, Romania; 7 Department of Cardiovascular Surgery, “Victor Babeș” University of Medicine and Pharmacy, 300041 Timisoara, Romania; 8 “Pius Brînzeu” Emergency County Clinical Hospital of Timisoara, 300723 Timisoara, Romania

**Keywords:** cardiac resynchronization therapy, fusion pacing, super-response, CRT

## Abstract

**Background/Objectives**: Cardiac resynchronization therapy (CRT) with fusion pacing (“LV only”), also known as fusion-CRT (f-CRT), represents a feasible alternative to cardiac resynchronization therapy (CRT) with biventricular pacing (BiVP), not only in cases of BiVP failure, but also as a primary therapy option due to its potential benefits over traditional CRT. Fusion pacing may be particularly beneficial in selected patients and understanding the structural and functional differences between responders could guide future optimization strategies. This study provides a descriptive comparison between super-responders (SRs) and non-super-responders (NSRs) undergoing fusion-CRT. **Methods**: Patients with RA/LV-only pacing systems or biventricular CRT systems operating predominantly in LV-only pacing mode due to intrinsic RV conduction were included. A follow-up protocol was conducted for all patients at 6 months and then annually. Data from the most recent follow-up were used for statistical analysis. Super-responders (SRs) were those with substantial reverse remodeling, quantified by a ≥30% reduction in LVESV and a stable LVEF of ≥45% at follow-up. Although SRs were defined based on these reverse remodeling criteria, separate analyses of additional echocardiographic parameters (e.g., left atrial dimensions) were performed to independently assess the broader impact of fusion-CRT on cardiac structure and function. **Results**: Among 71 patients, 55 were non-super-responders (NSRs) and 16 were super-responders (SRs), with a mean follow-up of 43.2 months. SRs were predominantly female and had smaller left ventricular (LV) dimensions: LVEDd (6.30 cm vs. 6.80 cm, *p* = 0.02), LVEDV (185 mL vs. 240 mL, *p* = 0.03), LVESV (132.5 mL vs. 175 mL, *p* = 0.03), and a higher LVEF (*p* = 0.03). The follow-up LVEF was positively correlated with changes in LVESV (ρ = 0.557, *p* < 0.001), but not with NYHA class changes (ρ = 0.184, *p* = 0.125). Larger baseline LV and left atrial (LA) volumes were associated with a reduced follow-up LVEF (LVESV: ρ = −0.426, *p* < 0.001; LVEDV: ρ = −0.394, *p* < 0.001; LAv: ρ = −0.374, *p* = 0.001). Both groups showed improvement in the NYHA class (*p* < 0.001, *p* = 0.007). MR improved significantly in SRs (*p* = 0.02) and worsened slightly in NSRs (*p* = 0.13), while TR worsened significantly in the NSRs group (*p* = 0.03). **Conclusions**: Our findings highlight key differences in clinical and echocardiographic parameters between SRs and NSRs following fusion-CRT. These observations may contribute to a better understanding of response patterns and inform future prospective studies aiming to optimize patient selection and timing of therapy.

## 1. Introduction

Cardiac resynchronization therapy (CRT) has revolutionized heart failure treatment, specifically for patients with left ventricular (LV) systolic dysfunction and left bundle branch block (LBBB). Since the early stages of research into this intervention, fusion pacing has been explored as an alternative to standard CRT, aiming to improve excitation–contraction coupling and deliver better hemodynamic outcomes [1,2].

It has been observed that right ventricular (RV)-only pacing disrupts LV intrinsic stimulation, causing a prolonged activation time and less efficient ventricular synchrony, impacting cardiac output [3]. The fusion pacing strategy combines LV pacing with intrinsic LV excitation through the right bundle branch, resulting in a more physiological activation pattern than biventricular pacing (BiVP) and overall superior resynchronization [3,4,5]. Fusion pacing has been demonstrated to be equally advantageous as BiVP for left ventricular systolic function in acute hemodynamic studies [1,6,7], in long-term follow-up studies [8,9,10], and even in circumstances where LV pacing is unlikely to result in the fusion of two activation wavefronts induced by LV pacing and intrinsic conduction [7,11]. A need for an RV lead in CRT comes primarily from technical considerations in cases where CRT-D is mandatory for preventing sudden cardiac death or in patients with atrioventricular block or persistent atrial fibrillation (AF).

Despite meeting guideline-based inclusion criteria, approximately one-third of patients undergoing CRT fail to achieve the expected benefits, such as improved cardiac function and symptom relief [12,13]. In contrast, approximately 20–30% of patients who experience a significant change in cardiac structure and function, with important reverse remodeling, are typically known as “super-responders”. This heterogeneity in response has led to a growing interest in identifying predictors of response or alternative resynchronization strategies, such as using either f-CRT or physiological pacing [14,15].

Data regarding f-CRT remain limited, as this approach has been relatively neglected, perhaps due to the earlier focus on standard CRT. This study aims to characterize super-response in f-CRT by comparing follow-up findings between NSRs and SRs, focusing on echocardiographic and clinical differences observed during long-term follow-up, rather than establishing predictive criteria.

## 2. Materials and Methods

### 2.1. Study Design and Study Population

This retrospective cohort study analyzed data from 71 consecutive patients with symptomatic heart failure who were implanted in a single center between September 2009 and September 2024. The study population consisted of candidates for CRT who received RA/LV-only pacing systems or biventricular CRT systems operating predominantly in LV-only pacing mode. All patients received implantations using a direct transcutaneous puncture technique with left ventricular (LV) lead placement. In cases where this approach was unsuccessful due to anatomical considerations, the implantation was not performed, and alternative pacing strategies were attempted instead.

Patients were followed up according to the hospital’s standard protocol, with reevaluation after 6 months and then annually. Data from the most recent follow-up were used for statistical analysis.

The study protocol was approved by our Research Ethics Committee, and all patients provided informed consent for device placement and study participation. The study complies with the principles outlined in the Declaration of Helsinki.

### 2.2. Inclusion Criteria

F-CRT was performed based on the following inclusion criteria: heart failure patients with NYHA class II-IV who remained symptomatic despite three months of optimal medical treatment [3,16,17], a sinus rhythm with normal atrioventricular (AV) conduction, a reduced left ventricular ejection fraction (LVEF) ≤ 35%, and a QRS duration of ≥130 ms. Some patients received implantations under the earlier guideline recommendations with a QRS duration ≥ 120 ms.

QRS duration was manually measured from standard 12-lead electrocardiograms using digital calipers by two experienced cardiologists, with the lead exhibiting the clearest QRS morphology selected for analysis; discrepancies were resolved by consensus. Although initial statistical outputs displayed values with two-decimal precision, all QRS duration data have been rounded to the nearest whole number to accurately reflect the measurement’s inherent resolution.

### 2.3. Exclusion Criteria

Exclusion criteria for the f-CRT pacing strategy were as follows: heart failure patients with NYHA class I and patients who required BiVP with permanent AF or total AV block. Patients with insufficient data were also excluded.

Additionally, 2 patients who showed no clinical response were excluded from the analysis, and no separate group was formed due to their small number. Patients who received implantations with alternative pacing strategies (such as left bundle branch pacing [LBBP] or epicardial lead placement) were also excluded.

### 2.4. Device Programming in F-CRT

Based on the existing literature, the “fusion band” was defined as the range of AV intervals during which the surface ECG (mainly in lead V1) displayed an intermediate morphology between native LBBB and paced right bundle branch block patterns [14,18].

The programming protocol consisted of device interrogation at 24 h post-implant, at discharge, and with every follow-up visit. At admission, all devices were programmed with a resting heart rate of 60 beats per minute (bpm) and a maximum tracking rate (MTR) of 130 bpm. Adapted AV intervals were adjusted to obtain proper fusion capture, with the dynamic AV interval at baseline set equal to the sensed AV interval, allowing fusion pacing. A 12-lead electrocardiogram (ECG) was recorded during each interrogation in scenarios with pacing turned on or off. Device optimization with every follow-up visit included titration of beta-blockers and/or ivabradine dose to achieve stability of the spontaneous PR interval.

### 2.5. Criteria for Response and Study Groups

Patients were divided into 2 groups based on their response to f-CRT: super-responders (SRs) and non-super-responders (NSRs). SRs were defined as patients with significant reverse remodeling, quantified by a ≥30% reduction in left ventricular end-systolic volume (LVESV) and a stable left ventricular ejection fraction (LVEF) of ≥45% at follow-up. NSRs were defined as patients who showed some clinical improvement but did not fulfill the definition of super-response. In our cohort, only 2 patients showed no clinical response at all. Due to their low number, these patients were not analyzed as a separate group and were excluded. Therefore, the NSRs group primarily includes patients with a partial response, and the overall study population consisted of responders, indicating the careful patient selection and implantation strategy employed in our center.

In addition, we acknowledge that analyses involving these parameters are inevitably influenced by the response definition itself. Therefore, we conducted an independent analysis of additional remodeling parameters to ensure an unbiased evaluation of fusion-CRT effects.

### 2.6. Echocardiographic Assessment

All patients were evaluated before the procedure using a VIVID echocardiograph (GE Health Medical, Milwaukee, WI, USA) with a 2.5 MHz transducer, following standard techniques and including simultaneous ECG recordings. Two-dimensional echocardiography was used to measure interventricular septum diameter (IVSd), posterior wall diameter (PWLVd), LV end-diastolic diameter (LVEDd), LV end-diastolic and end-systolic volumes (LVEDV, LVESV), LV ejection fraction (LVEF) calculated by using Simpson’s biplane method, and left atrium (LA) quantification, such as LA diameter (LAd), LA area (LAa), and LA volume (LAv and LAVI). The maximal atrial size was considered, and all left atrial measurements were performed during end-systole, right before the mitral valve opening. Furthermore, functional factors such as mitral valve regurgitation (MR) and/or tricuspid valve regurgitation (TR) were evaluated and categorized as mild, moderate, or severe. Other parameters included systolic pulmonary arterial pressure (sPAP) and the E/A ratio to analyze the hemodynamic and structural state of the heart.

### 2.7. Statistical Analysis

In this study, data are presented as mean ± standard deviation (SD) for the variables with a normal distribution, and as medians and interquartile ranges (IQRs) for the variables that have a non-Gaussian distribution. The categorical variables are presented as counts and percentages. To assess the normality of the data, the Shapiro–Wilk test was performed, and for the evaluation of the differences between continuous variables, we used Mann–Whitney U tests. For the categorical variables, we applied the chi-squared tests or Fisher’s exact tests. Spearman’s rank correlation was applied to evaluate the relationships between the numerical variables, and longitudinal trends were determined by utilizing Wilcoxon signed-rank tests for the continuous variables. We used McNemar’s test for the paired categorical data. Logistic regression was applied to predict super-responders. Linear regression analyzed LVEF and NYHA changes. Interaction terms were used to evaluate the combined influence of QRS length on LVEF improvement.

Two derived variables were introduced (Change LVES and Change NYHA) to quantify improvements in LVESV and NYHA functional class. Change LVESV is defined as the absolute difference between baseline and follow-up LVESV, meaning that higher values reflect greater reverse remodeling. Similarly, Change NYHA measures differences between the baseline and the follow-up NYHA class distribution, and functional improvement was indicated by negative values in this variable. All data were analyzed using R (version 4.3.0; R Core Team, 2023) and RStudio (version 2023.06.0+421; RStudio Team, 2023).

## 3. Results

### 3.1. Baseline Characteristics of Fusion-CRT in Non-Super-Responders and Super-Responders

#### 3.1.1. Baseline Clinical Characteristics, Comorbidities, and Follow-Up

**Figure A1:**
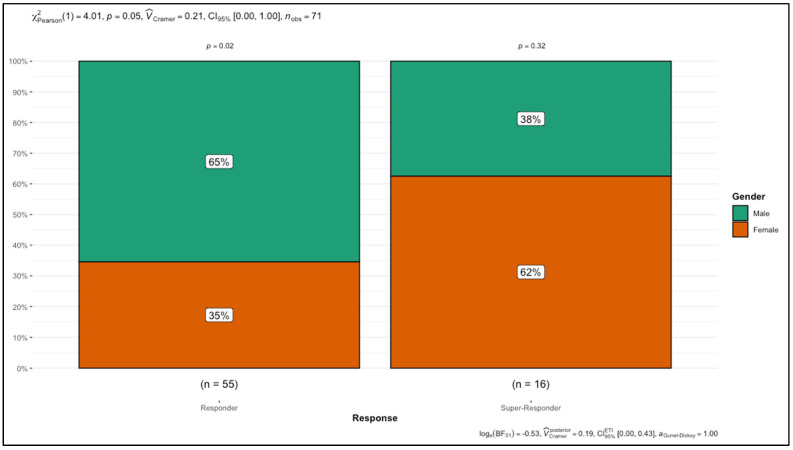
Sex distribution among NSRs and SRs to f-CRT.

The study population included 55 NSRs and 16 SRs. There was no significant difference between NSRs and SRs in terms of age (*p* = 0.75), body mass index (BMI) (*p* = 0.94), body surface area (BSA) (*p* = 0.45), or QRS duration (*p* = 0.31). Sex distribution reached statistical significance, with most of the NSRs being males (65.45%) and most of the SRs being females (62.5%; *p* = 0.05) (Table 1). The distribution of patients by sex based on their response to f-CRT is illustrated in Figure A1 (Appendix A).

Arterial hypertension (AHT) prevalence did not differ between SRs (68.75%) and NSRs (61.82%; *p* = 0.61). Heart failure etiology (*p* = 0.85) and chronic kidney disease (CKD) stages were comparable between the two groups (*p* = 0.21), even though SRs had a higher percentage of patients with milder CKD stages (G1–G2) and no patients had severe CKD (G4). Patients were followed up for a mean period of 43.2 months, with no significant variations in follow-up time between NSRs and SRs (*p* = 0.49).

#### 3.1.2. Baseline Echocardiographic Parameters

**Figure 1:**
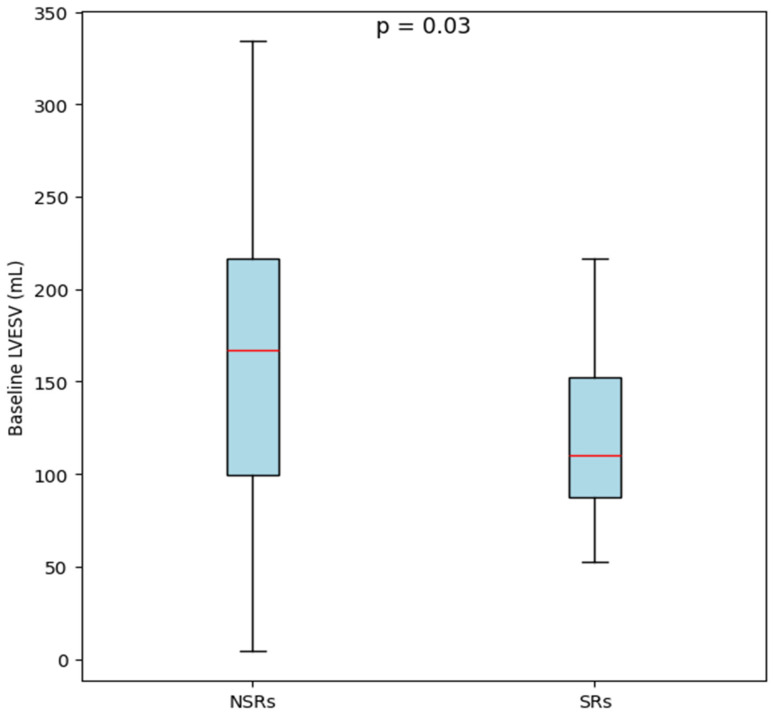
Comparison of baseline left ventricular end-systolic volume between non-super-responders and super-responders.

IVSd (*p* = 0.43) and PWLVd measurements (*p* = 0.39) did not significantly differ across the two groups (Table 2). SRs had lower values of left ventricular dimensions: LVEDd (median 6.30 cm [IQR: 5.30–6.73] vs. 6.80 cm [IQR: 6.20–7.30], *p* = 0.02), LVEDV (median 185.00 mL [IQR: 146.50–224.75] vs. 240.00 mL [IQR: 193.50–300.00], *p* = 0.03), and LVESV (median 132.50 mL [IQR: 99.50–162.00] vs. 175.00 mL [IQR: 130.00–231.00], *p* = 0.03). Figure 1 illustrates the significant difference between the SRs and NSRs, representing that SRs had a consistently lower baseline LVESV. In addition, SRs had a significantly higher mean LVEF compared to NSRs (*p* = 0.03), meaning better baseline systolic function was observed in the SR group.

Left atrial parameters were also reduced in SRs, such as LAa (median 25.50 cm^2^ [IQR: 20.48–26.91] vs. 26.91 cm^2^ [IQR: 26.45–27.00], *p* = 0.02), LAv (median 78.50 mL [IQR: 49.75–102.50] vs. 99.72 mL [IQR: 80.50–129.00], *p* = 0.02), and LAVI (median 44.70 mL/m^2^ [IQR: 28.68–53.08] vs. 53.08 mL/m^2^ [IQR: 43.90–64.85], *p* = 0.03). On the contrary, the E/A ratio (*p* = 0.40) and systolic pulmonary arterial pressure (sPAP; *p* = 0.72) did not differ between NSRs and SRs. The baseline distributions of MR severity (*p* = 0.49) and TR severity (*p* = 0.78) were comparable across the two groups.

### 3.2. Correlation Analysis of Structural, Functional, and Electrical Parameters in f-CRT Outcomes

The correlation analysis (Table 3) confirmed a strong positive relationship between baseline and follow-up values for LVEF (ρ = 0.443, *p* < 0.001), LVESV (ρ = 0.603, *p* < 0.001), and LAv (ρ = 0.640, *p* < 0.001).

Since our SR definition is based on a ≥30% reduction in LVESV and a stable LVEF (≥45%) at follow-up, the significant correlations observed for LVEF and LVESV are anticipated by design. To address this and provide an independent evaluation of cardiac remodeling, we also analyzed other parameters, such as left atrial volume (LAv) and QRS duration, which are not part of the response criteria.

**Figure 2:**
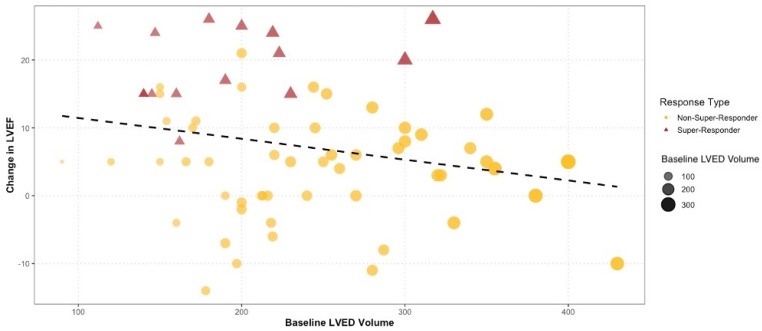
Impact of baseline left ventricle end-diastolic and end-systolic volumes on left ventricle ejection fraction.

The correlation analysis (Table 3) confirms a strong positive relationship between baseline and follow-up values for LVEF (ρ = 0.443, *p* < 0.001), LVESV (ρ = 0.603, *p* < 0.001), and LAv (ρ = 0.640, *p* < 0.001). As expected, follow-up LVEF was positively correlated with the percentage change in LVESV (ρ = 0.557, *p* < 0.001), further indicating that greater reductions in LVESV coincide with improved systolic function, while the correlation with the change in NYHA class did not reach statistical significance (ρ = 0.184, *p* = 0.125). In addition, significant inverse correlations were observed between follow-up LVEF and the baseline values of LVESV (ρ = −0.426, *p* < 0.001), LVEDV (ρ = −0.394, *p* < 0.001), and LAv (ρ = −0.374, *p* = 0.001), suggesting that patients with larger baseline chamber sizes experienced less of an improvement in systolic function—a trend further supported by the negative correlation between baseline LV volumes and the change in LVEF, as illustrated in Figure 2. Conversely, baseline QRS duration did not correlate significantly with follow-up LVESV (ρ = −0.169, *p* = 0.159) or LVEF (ρ = 0.201, *p* = 0.093), highlighting the importance of including independent remodeling parameters in our overall assessment.

### 3.3. Longitudinal Analysis of Structural and Functional Changes After Fusion-CRT

In our cohort, the NYHA class improved significantly in both groups (*p* < 0.001, *p* = 0.007; Table 4). Contrary to SRs, there was still a small proportion of NSRs remaining in class IV at follow-up. The severity of MR remained mostly unchanged. In NSRs, the MR worsened slightly, yet not significantly (*p* = 0.13), while in SRs, MR improved significantly, with a complete reduction in the severe MR (*p* = 0.02). In addition, TR worsened significantly in NSRs (*p* = 0.03), with a general trend towards increased severity. Table 4 provides a better overview of each subgroup.

**Figure 3:**
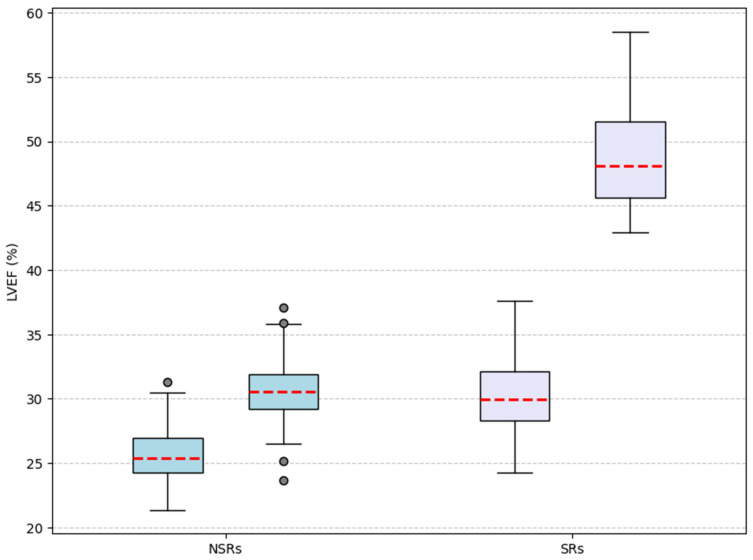
Change in left ventricular ejection fraction between baseline and follow-up.

Structural remodeling was more significant in SRs. LVEDV and LVESV remained constant in NSRs but demonstrated marked reductions in SRs (*p* < 0.001; Table 5). LVEF improved significantly in all patients, as illustrated in Figure 3. However, left atrial volume, indexed or not, remained unchanged. The E/A ratio decreased significantly only in SRs (*p* = 0.02). Systolic pulmonary arterial pressures decreased across the cohort (*p* < 0.001). Table 5 provides a detailed analysis of these results.

### 3.4. Univariate Logistic Regression Analysis of Predictors for the Super-Responder Status

In univariate logistic regression analyses, the baseline LVEDd (OR = 0.47, *p* = 0.023), left atrial volume (LAv; OR = 0.97, *p* = 0.015), and left atrial volume index (LAVI; OR = 0.96, *p* = 0.035) were significantly associated with SR status. It should be noted that our definition of a super-responder—based on a ≥30% reduction in LVESV and a stable LVEF of ≥45% at follow-up—might intrinsically influence these relationships. Consequently, while these baseline parameters appear predictive, their associations may be partly driven by the circularity in the study design, thus limiting a direct causal interpretation. Future studies with a prospective design and comprehensive multivariate models are required to further validate these associations. The Nagelkerke R^2^ values for these models ranged from 0.115 to 0.158. The results are presented in Table 6.

## 4. Discussion

Since its early stages, CRT has gained significant importance as a heart failure therapy for patients with electrical and mechanical dyssynchrony. BiVP is the standard strategy for obtaining ventricular resynchronization, improving mechanical performance, and reducing HF symptoms. However, around 30% of patients remain non-responders to CRT, motivating researchers to explore alternative pacing strategies. Fusion-CRT is especially advantageous for its potential to induce narrower-paced QRS complexes [19]. This observation is reinforced by recent studies that highlighted the idea that a wider-paced QRS duration is associated with a worse prognosis in terms of mortality [20].

Currently, the definition of super-response is inconsistent across various studies. Ypenburg et al. proposed to consider patients with a reduction in LVESV ≥ 30% at 6 months as SRs [21], Castellant et al. classified patients as SRs if they fulfilled two criteria, functional recovery and LVEF ≥ 50% [22], while Jin et al. described SRs as patients with a decrease in NYHA class ≥ 1, a two-fold or more increase in LVEF or a final LVEF ≥ 45%, and a reduction in LVESV ≥ 15% [23]. In our study, we investigated the potential predictors of super-response in f-CRT, and we defined it as a ≥30% reduction in LVESV along with a stable LVEF of ≥45% at follow-up.

Previous studies have identified several predictors of super-response in CRT, including widely accepted guideline recommendations such as LVEF < 35% and LBBB. In various studies, some other variables were associated with a better long-term response, such as sinus rhythm, female sex, non-ischemic etiology of HF, duration of HF symptoms, smaller LA and LV dimensions, milder mitral and tricuspid regurgitation, reduced BNP levels, or creatinine levels [16,17,24,25,26,27]. As there is limited research on this topic, results and observations from comparable studies with biventricular pacing will be referenced throughout this section.

It is important to note that the aim of our study was not to develop predictive models, but rather to describe the observed characteristics and clinical trajectories of patients classified as SRs versus NSRs. Our results suggest that demographic factors such as age, BMI, and BSA may not be key determinants in differentiating SRs from NSRs. However, the female sex reached statistical significance in predicting superior outcomes, as two-thirds of SRs were women. This aligns with previous studies, indicating that women may have a more favorable response to CRT due to hormonal influences in modulating the response to f-CRT, differences in cardiac structure, and conduction system physiology [28,29]. A possible explanation for these variations might be the increased degree of spontaneous fusion with intrinsic conduction observed in women, often attributed to shorter PR intervals [30]. The dependency of f-CRT on intrinsic conduction highlights the importance of optimization strategies like fusion-optimized intervals (FOIs), which may reduce these inconsistencies by precisely adjusting LV pacing in accordance with intrinsic RV conduction, leading to superior outcomes in male patients and bridging the gap in response rates between genders [31,32].

In our study, no changes in baseline QRS duration were observed between NSRs and SRs, which might explain the homogeneity in the outcomes of the two study groups. In addition, no significant correlation was found between baseline QRS duration and follow-up LVEF or LVESV. This can be attributed to the increased baseline QRS duration, over 150 ms, in most of the study patients.

Renal dysfunction is highly prevalent in patients with HF. It is known that between 25 and 50% of this population has a creatinine clearance under 60 mL/min/1.73 m^2^ [33,34,35]. A subanalysis of the MIRACLE study reported that patients with reduced eGFR experienced less significant reductions in LV dimensions [36], while Van Bommel et al. identified renal dysfunction as a predictor of echocardiographic non-response to CRT [37]. Our results are consistent with data from the literature. We observed that patients with advanced CKD had progressively worse LVEF improvements, recognizing the negative implications of renal dysfunction on f-CRT response. This may be caused by the interaction of inflammation, neurohormonal activation, vascular stiffness, and reduced myocardial perfusion [38]. In addition to its effects on remodeling, CKD also contributes to poor long-term survival in CRT patients [39,40].

Although earlier research demonstrated that the ischemic etiology of HF was associated with inferior CRT response [17], in our study, the ischemic cardiomyopathy did not significantly influence the probability of achieving SR status. This might be attributed to the limited number of ischemic patients, which was not a consequence of intentional selection bias. Our results may have corresponded more closely with the literature data if we had included a larger number of ischemic patients.

The baseline classification between NSRs and SRs becomes particularly significant when analyzing echocardiographic data, correlating the SR status with smaller baseline LA dimensions. Even though the baseline LAa, LAv, and LAVI were smaller at admission, the E/A ratio did not differ in our cohort. According to the univariate regression model, baseline LAv accounted for 15.8% and LAVI accounted for around 11.5% of the variability in SR status, underscoring the importance of baseline LA size in predicting response to f-CRT. These results correspond with a meta-analysis that demonstrated the relationship between reduced baseline LAVI and superior response to traditional CRT by defining a threshold of 34 mL/m^2^ [41]. Cardiac resynchronization therapy can reduce LA pressure and wall tension by limiting the stretch-induced neurohormonal activation, collagen deposition, and interstitial fibrosis, therefore contributing to atrial reverse remodeling [42]. However, some data indicate that atrial remodeling may progress more slowly after traditional CRT [43,44].

Based on our results, MR significantly decreased among SRs and remained unchanged in NSRs, which could represent a cause for limiting the LA unloading and size reduction. Evidence shows that a decrease in MR severity is associated with an increase in LA reservoir strain [45,46]. According to Stassen et al. [42], approximately one-fourth of CRT patients reached complete atrial and ventricular reverse remodeling. This is correlated with superior long-term survival compared to patients who experience only a partial response to traditional CRT. In our cohort, the lack of a significant change in LA volumes and MR degree may reflect a more pronounced substrate for fibrosis that originated from an advanced structural change in the heart, which in turn limits the potential for meaningful reverse remodeling.

TR progressed differently between the study groups, with significant worsening only for NSRs. Even though SRs had a trend towards improvement, this was not statistically significant. The TR degree seems to be dependent on right heart remodeling, which may appear after the left heart recovery because of persistent LV and LA dysfunction, elevated residual sPAP, or severe tricuspid annular dilatation [47]. Moreover, right heart recovery appears to happen in conjunction with left heart reverse remodeling and is also influenced by baseline parameters such as LVEF, QRS duration, or age [48,49]. In our study, sPAP significantly decreased after f-CRT, with the most substantial reductions seen in SRs. This indicates that better hemodynamics led to better ventricular–arterial coupling and reduced pulmonary vascular resistance for all patients following f-CRT, even though it was not correlated with a decrease in TR severity. The persistence of elevated pressures in certain patients may suggest the existence of residual right heart dysfunction or a delay in RV reverse remodeling. Both baseline values, as well as the degree of improvement in parameters such as RV size, RV function, and TR degree, have significant prognostic value for CRT patients and are associated with long-term survival [50].

**Figure A2:**
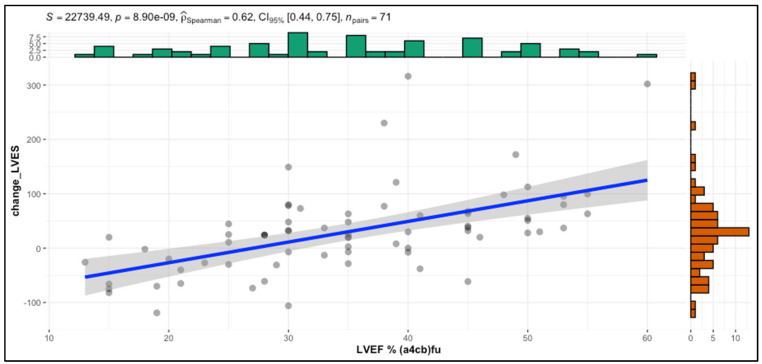
Greater reductions in LVESV were associated with improved LVEF.

In our study, we demonstrated that baseline LVEDV and LVESV were negatively correlated with follow-up LVEF (Table 3). Smaller baseline LV volumes, particularly LVED and LVESV, were associated with superior outcomes, especially in terms of LVEF improvement (Table 2, Figure 2). Patients with less severe heart failure and less cardiac structural damage are ideal candidates for resynchronization therapy due to the presence of contractile reserve [51]. It is known that LVESV reduction is a strong indicator for LV reverse remodeling and has a strong correlation with LVEF improvement (Figure A2). However, long-term survival data on this issue found that even some patients with significant LV dilatation can show reverse remodeling, and those that respond also have increased survival [52].

Taken individually, LVEDd explains approximately 12.6% of the variance in SR outcomes. In addition, PWLVd was demonstrated to be a strong positive predictor of LVEF improvement, while thicker IVSd was associated with a worse response. This may reflect the complexity of LV geometry and the influence of regional myocardial function on cardiac output. Furthermore, LV reverse remodeling is significantly associated with increased survival, highlighting its prognostic importance [52,53].

During this study, patients experienced significant changes in their NYHA functional class, proving the efficacy of f-CRT in reducing the symptoms associated with heart failure. However, we have demonstrated that follow-up LVEF did not correlate with NYHA class variations, suggesting that symptomatic relief may not consistently correspond with changes in systolic function.

Overall, these findings suggest that a thorough assessment of baseline structural and functional status, as well as a complete screening for comorbidities, is essential for optimizing f-CRT outcomes. In addition, current progress, such as left bundle branch-optimized cardiac resynchronization therapy (LOT-CRT), supports the concept of f-CRT by using conduction system pacing with conventional LV pacing to improve CRT. LOT-CRT can be applied selectively, capturing only the left bundle—or non-selectively, stimulating both the conduction system and adjacent myocardium, with both pacing strategies showing favorable effects on improving electrical synchronization and clinical outcomes [54], strengthening the potential advantages of reconsidering native conduction-based strategies in CRT programming. Further research is necessary to guide patient selection by developing predictive models that integrate these parameters more accurately.

The limitations of this study come from its retrospective design and single-center setting, which resulted in a restricted sample size. Based on this, the findings from our analysis should be interpreted as observational associations rather than causal predictors. Some patients and variables could not be included due to missing data or unreliable collection. Larger prospective studies are needed to validate these findings and refine predictive models for f-CRT response.

In addition, another limitation is the potential bias introduced by our response definition. Since SR status is determined by reverse remodeling criteria (i.e., LVESV reduction and stable LVEF), the predictive associations observed in our logistic regression analysis using baseline LV and LA measurements may be influenced by its intrinsic circularity. Furthermore, the trends which were noted among baseline LA and LV volumes require prospective validation in larger cohorts before clinical guidance can be derived. This caveat highlights the necessity for future prospective studies that incorporate robust multivariate models to establish causality more definitively between baseline characteristics and response to fusion-CRT.

## 5. Conclusions

While highlighting key differences in clinical and echocardiographic parameters between SRs and NSRs patients following fusion-CRT, our study provides a comprehensive comparison of structural and functional changes among this category of patients, with findings that suggest that early-stage HF and less extensive baseline remodeling are associated with greater improvements. These observations may contribute to a better understanding of response patterns and inform future prospective studies aiming to optimize patient selection and timing of therapy.

## Figures and Tables

**Table 1:** Baseline demographics and comorbidities of non-super-responders and super-responders to fusion-CRT.

	Non-Super-Responder	Super-Responder	*p*-Value
Variable	*N* = 55	*N* = 16	
**Baseline clinical characteristics**			
Age [years]	70.0 (62.0–74.5)	67.5 (65.0–71.5)	0.75
Sex			
Male	36 (65.45%)	6 (37.5%)	0.05 *
Female	19 (34.55%)	10 (62.5%)	
Body mass index [kg/m^2^]	28.30 (25.85–32.60)	27.90 (26.73–31.40)	0.94
Body surface area [m^2^]	1.90 (1.80–2.00)	1.80 (1.78–2.00)	0.45
QRS duration [ms]	160.0 (150.0–168.0)	160.0 (146.0–160.0)	0.31
**Comorbidities**			
Arterial hypertension [yes]	34 (61.82%)	11 (68.75%)	0.61
Chronic kidney disease			
G1	9 (16.36%)	5 (31.25%)	0.21
G2	15 (27.27%)	7 (43.75%)	
G3a	13 (23.64%)	3 (18.75%)	
G3b	17 (30.91%)	1 (6.25%)	
G4	1 (1.82%)	0 (0%)	
Heart failure etiology			
Ischemic	6 (10.91%)	1 (6.25%)	0.85
Non-ischemic	9 (16.36%)	3 (18.75%)	
Idiopathic	40 (72.73%)	12 (75%)	
**Follow-up**			
Mean follow-up [months]	43.3	42.9	0.49

Data presented as median and interquartile range (IQR) or *n* (%). Variables and abbreviations: chronic kidney disease (CKD) stages are classified according to KDIGO (Kidney Disease: Improving Global Outcomes) based on estimated glomerular filtration rate (eGFR, mL/min/1.73 m^2^): G1 (≥90), G2 (60–89), G3a (45–59), G3b (30–44), and G4 (15–29). Heart failure is classified as ischemic (coronary artery disease-related), non-ischemic (non-coronary causes), or idiopathic (unknown cause). Statistical analysis: Mann–Whitney U test was used for continuous variables (e.g., age, BMI, QRS duration), and Pearson’s chi-squared test was used for categorical variables (e.g., gender, comorbidities). Fisher’s exact test was applied where necessary. Statistical significance: * *p* < 0.05.

**Table 2:** Baseline echocardiographic characteristics of non-super-responders and super-responders.

		Non-Super-Responder	Super-Responder	*p*-Value
Variable		*N* = 55	*N* = 16	
**Echocardiographic data at baseline**				
Interventricular septum wall diameter [cm]		1.10 (1.00–1.25)	1.05 (1.00–1.22)	0.43
Left ventricle posterior wall diameter [cm]		1.10 (1.00–1.20)	1.15 (1.00–1.20)	0.39
Left ventricle end-diastolic diameter [cm]		6.80 (6.20–7.30)	6.30 (5.30–6.73)	0.02 *
Left ventricle end-diastolic volume [mL]		240.00 (193.50–300.00)	185.00 (146.50–224.75)	0.03 *
Left ventricle end-systolic volume [mL]		175.00 (130.00–231.00)	132.50 (99.50–162.00)	0.03 *
Left ventricle ejection fraction [%]		26 ± 6.9	29.4 ± 4.5	0.03 *
Left atrium area [cm^2^]		26.91 (26.45–27.00)	25.50 (20.48–26.91)	0.02 *
Left atrium volume [mL]		99.72 (80.50–129.00)	78.50 (49.75–102.50)	0.02 *
Left atrium volume index [mL/m^2^]		53.08 (43.90–64.85)	44.70 (28.68–53.08)	0.03 *
E/A ratio		1.50 (0.72–2.00)	1.15 (0.57–1.63)	0.4
Systolic pulmonary arterial pressure [mmHg]		46.29 (41.50–49.00)	46.29 (46.29–50.00)	0.72
Mitral valve regurgitation				0.49
	Mild	11 (20%)	5 (31.3%)	
	Moderate	33 (60%)	8 (50%)	
	Severe	11 (20%)	3 (18.8%)	
Tricuspid valve regurgitation				0.78
	Mild	23 (41.8%)	7 (43.8%)	
	Moderate	30 (54.5%)	9 (56.3%)	
	Severe	2 (3.6%)	0	

Data presented as mean ± standard deviation (for normally distributed variables), median and interquartile range (IQR) (non-normally distributed variables), or *n* (%). E/A ratio represents the early (E-wave) to late (A-wave) diastolic filling velocity ratio. Statistical analysis: Mann–Whitney U test was used for continuous variables, while Pearson’s chi-squared or Fisher’s exact test were used for categorical variables. Statistical significance: * *p* < 0.05.

**Table 3:** Correlation analysis of baseline and follow-up variables in fusion-CRT outcomes.

Correlation	Variable 1	Variable 2	ρ (rho)	*p*-Value
Correlations between baseline variables and follow-up variables	LVEF (b)	LVEF (fu)	0.443	<0.001 **
	Left ventricle end-systolic volume (b)	Left ventricle end-systolic volume (fu)	0.603	<0.001 **
	Left atrium volume (b)	Left atrium volume (fu)	0.640	<0.001 **
Correlations using left ventricle ejection fraction	LVEF (fu)	Change left ventricle end-systolic volume	0.557	<0.001 **
	LVEF (fu)	Change NYHA	0.184	0.125
	LVEF (fu)	Left ventricle end-systolic volume (b)	−0.426	<0.001 **
	LVEF (fu)	Left ventricle end-diastolic volume (b)	−0.394	<0.001 **
	LVEF (fu)	Left atrium volume (b)	−0.374	0.001 **
Correlations between baseline QRS duration and left ventricle reverse-remodeling parameters	QRS duration (b)	Left ventricle end-systolic volume (fu)	−0.169	0.159
	QRS duration (b)	LEF (fu)	0.201	0.093

Data presented as: Spearman’s correlation coefficient (ρ) and *p*-values for statistical significance. Variables and abbreviations: (b)—baseline values; (fu)—follow-up values; LVEF—left ventricular ejection fraction; change LVES—percentage change in left ventricular end-systolic volume from baseline to follow-up; change NYHA—numerical change in New York Heart Association functional class from baseline to follow-up. Statistical analysis: Spearman’s rank correlation test, with ρ (rho) as the correlation coefficient. Statistical significance: ** *p* < 0.01.

**Table 4:** Longitudinal changes in functional status and valvular regurgitations after f-CRT.

	All Patients (*N* = 71)		*p*-Value	Non-Super-Responders (*N* = 55)		*p*-Value	Super-Responders (*N* = 16)		*p*-Value
Variable	Baseline	Follow-Up		Baseline	Follow-Up		Baseline	Follow-Up	
New York Heart Association Class			<0.001 **			<0.001 **			0.007 **
I	0%	8.5%		0%	7.3%		0%	12.5%	
II	50.7%	59.1%		49.1%	54.5%		56.3%	75%	
III	45.1%	23.9%		45.5%	27.3%		43.8%	12.5%	
IV	4.2%	8.5%		5.5%	10.9%		0%	0%	
Mitral valve regurgitation			0.99			0.13			0.02 *
Mild	22.5%	29.6%		20%	20%		31.3%	62.5%	
Moderate	57.7%	43.7%		60%	45.5%		50%	37.5%	
Severe	19.7%	26.8%		20%	34.5%		18.8%	0%	
Tricuspid valve regurgitation			0.2			0.03 *			0.15
Mild	42.3%	42.3%		41.8%	34.5%		43.8%	68.8%	
Moderate	54.9%	43.7%		54.5%	47.3%		56.3%	31.3%	
Severe	2.8%	14.1%		3.6%	18.2%		0%	6.3%	

Data presented as percentages (%). Percentages represent the proportion of patients in each category. Variables and abbreviations: ALL—all patients in the study, *n* = 71; NSR—non-super-responders, *n* = 55; SR—super-responders, *n* = 16. Statistical analysis: Wilcoxon signed-rank test was used for paired comparisons. Statistical significance: * *p* < 0.05; ** *p* < 0.01.

**Table 5:** Longitudinal echocardiographic changes after f-CRT.

	All Patients (*N* = 71)		*p*-Value	Non-Super-Responders (*N* = 55)		*p*-Value	Super-Responders (*N* = 16)		*p*-Value
Variable	Baseline	Follow-Up		Baseline	Follow-Up		Baseline	Follow-Up	
Left Ventricle End-Diastolic Volume [mL]	220	195	0.03 *	240	219.7	0.8	185	117.5	<0.001 **
Left Ventricle End-Systolic Volume [mL]	160	130	0.002 *	175	162	0.3	132.5	65.2	<0.001 **
Left Ventricle Ejection Fraction [%]	26.7	35	<0.001 **	26	30.7	<0.001 **	29.4	50.1	<0.001 **
Left Atrium Volume [mL]	96	93	0.76	99.7	102	0.2	78.5	66.5	0.06
Left Atrium Volume Index [mL/m^2^]	52.9	54.7	0.66	53.1	54.7	0.3	44.7	38.4	0.2
E/A Ratio	1.5	0.8	0.06	1.5	1.3	0.2	1.2	0.7	0.02 *
Systolic Pulmonary Arterial Pressure [mmHg]	46.3	39.8	<0.001 **	46.3	39.8	0.005 *	46.3	37.4	0.02 *

Data presented as mean ± standard deviation (for normally distributed variables), median and interquartile range (IQR) (non-normally distributed variables) or *n* (%). Variables and abbreviations: E/A ratio represents the early (E-wave) to late (A-wave) diastolic filling velocity ratio. Statistical tests: Wilcoxon signed-rank test or paired samples T-test were used for paired comparisons between baseline and follow-up measurements. *p*-value—probability value for statistical hypothesis testing. Statistical significance: * *p* < 0.05; ** *p* < 0.01.

**Table 6:** Univariate logistic regression analysis of predictors for super-responder status.

Predictors	Odds Ratios	Confidence Interval	*p*-Value	R^2^ Nagelkerke
Left ventricle end-diastolic diameter (b) [cm]	0.47	0.23–0.86	0.023 *	0.126
Left atrium volume (b) [mL]	0.97	0.95–0.99	0.015 *	0.158
Left atrium volume index(b) [mL/m^2^]	0.96	0.92–0.99	0.035 *	0.115

Data presented as Odds Ratios with 95% Confidence Intervals and *p*-values for statistical significance; *p*-value—probability value for statistical hypothesis testing for Wald’s test; R^2^ Nagelkerke—Nagelkerke’s pseudo-R-squared value, representing the proportion of variance explained by the model. Statistical significance: * *p* < 0.05. b is “baseline”.

## Data Availability

Data are available on request due to restrictions (privacy and ethical reasons).
